# Access to general practice and visits to accident and emergency departments in England: cross-sectional analysis of a national patient survey

**DOI:** 10.3399/bjgp14X680533

**Published:** 2014-06-30

**Authors:** Thomas E Cowling, Matthew J Harris, Hilary C Watt, Daniel C Gibbons, Azeem Majeed

**Affiliations:** Department of Primary Care and Public Health, Imperial College London, London.; Department of Primary Care and Public Health, Imperial College London, London.; Department of Primary Care and Public Health, Imperial College London, London.; Department of Primary Care and Public Health, Imperial College London, London.; Department of Primary Care and Public Health, Imperial College London, London.

**Keywords:** access to health care, emergency departments, general practice, patient appointments, primary health care, urgent care

## Abstract

**Background:**

The annual number of unplanned attendances at accident and emergency (A&E) departments in England increased by 11% (2.2 million attendances) between 2008–2009 and 2012–2013. A national review of urgent and emergency care has emphasised the role of access to primary care services in preventing A&E attendances.

**Aim:**

To estimate the number of A&E attendances in England in 2012–2013 that were preceded by the attending patient being unable to obtain an appointment or a convenient appointment at their general practice.

**Design and setting:**

Cross-sectional analysis of a national survey of adults registered with a GP in England.

**Method:**

The number of general practice consultations in England in 2012–2013 was estimated by extrapolating the linear trend of published data for 2000–2001 to 2008–2009. This parameter was multiplied by the ratio of attempts to obtain a general practice appointment that resulted in an A&E attendance to attempts that resulted in a general practice consultation estimated using the GP Patient Survey 2012–2013. A sensitivity analysis varied the number of consultations by ±12% and the ratio by ±25%.

**Results:**

An estimated 5.77 million (99.9% confidence interval = 5.49 to 6.05 million) A&E attendances were preceded by the attending patient being unable to obtain a general practice appointment or a convenient appointment, comprising 26.5% of unplanned A&E attendances in England in 2012–2013. The sensitivity analysis produced values between 17.5% and 37.2% of unplanned A&E attendances.

**Conclusion:**

A large number of A&E attendances are likely to be preceded by unsuccessful attempts to obtain convenient general practice appointments in England each year.

## INTRODUCTION

The annual number of unplanned attendances at accident and emergency (A&E) departments in England increased by 11% (2.2 million attendances) between 2008–2009 and 2012–2013.^1^ Planned follow-up attendances, for the removal of sutures for example, are excluded here (approximately 2% of attendances^2^).^1^

A national review of urgent and emergency care has emphasised the role of access to primary care services, including general practice, in preventing A&E attendances.^3^ In April 2014, the UK Department of Health revealed the details of a pilot scheme that will see 1147 general practices in England, with approximately 7.5 million registered patients, begin to offer appointments outside of current opening hours.^4^ When this scheme was first announced, the government stated that the pilot was seen as ‘the first step’ to rolling the policy out across the country, expecting that it could reduce utilisation of A&E services.^5^ These proposals have been controversial^6^ and may not be feasible without significant improvements in funding for general practice services.^7^ The Official Opposition to the current government, the Labour Party, have pledged to ensure patients are able to see a GP within 48 hours if elected, again presuming that this will relieve pressure on A&E departments.^8^

A national, cross-sectional analysis of 7856 practices in England concluded that practices providing ‘more timely’ access to care had lower rates of A&E attendances in 2010–2011 after adjustment for the characteristics of practices’ registered populations. This analysis focused on self-referred emergency department attendances that ended in the patient being discharged.^9^ The mechanism presumed to underlie this association is that some patients who are unable to obtain a general practice appointment, or one that they consider timely, subsequently visit an A&E department. Although this has been confirmed through interviews with patients that have attended an A&E department,^10^ the frequency with which it occurs and its consequent relevance to national policy remain unknown.

This study was an exploratory analysis that estimated the number of A&E attendances in England in 2012–2013 that were preceded by the attending patient being unable to obtain an appointment or a convenient appointment at their general practice.

## METHOD

The analysis used two parameters, both estimated for England for the 2012–2013 financial year: the number of general practice consultations, and the ratio of attempts to obtain a general practice appointment that resulted in an A&E attendance to attempts that resulted in a general practice consultation. These parameters were multiplied to obtain the final estimate.

**How this fits in**

Previous research suggests that general practices providing ‘more timely’ access to care have lower rates of A&E attendances in England. It was unknown how often patients who are unable to obtain a convenient general practice appointment subsequently visit an A&E department. It is estimated that the frequency of such attendances in England in 2012–2013 was 5.77 million: 26.5% of unplanned A&E attendances in this year.

### Number of general practice consultations

The annual number of general practice consultations has previously been estimated for a 14-year period up to 2008–2009 using the QResearch^®^ database.^11^ The estimates increase linearly from 2000–2001 (225.3 million) to 2008–2009 (303.9 million).^11^ Simple linear regression was used to predict the number of consultations in 2012–2013, under the assumption that the linear trend observed between 2000–2001 and 2008–2009 continued through to 2012–2013. Face-to-face and telephone consultations with GPs and nurses are included in this estimate.

### Ratio of relevant A&E attendances to general practice consultations

The national weighted results of the GP Patient Survey (GPPS) 2012–2013 were used to calculate the second parameter required. All general practices in England with eligible patients (aged ≥18 years old, GP-registered, and with a valid NHS number) were included in the survey (*n* = 8169).^11^ Questionnaires were sent to 2 761 123 patients, with a response rate of 35.2% (*n* = 971 232).^12^ A weighting scheme, applied to the results to account for differential response patterns, adjusted for the age, sex, ethnicity, deprivation, marital status, employment status, region of England, and other characteristics of responders and their residential areas.^12^ This weighting scheme also ensured the weighted sample of responders from each general practice resembled their eligible populations.^12^ Consequently, the weighted age–sex distribution of responders was very similar to the age–sex distribution of eligible patients nationally.^12^ Questions included in previous versions of the GPPS relating to access to care have demonstrated construct validity and reliability,^13^,^14^ and the questionnaire has undergone testing in cognitive interviews.^15^

**Figure 1:**
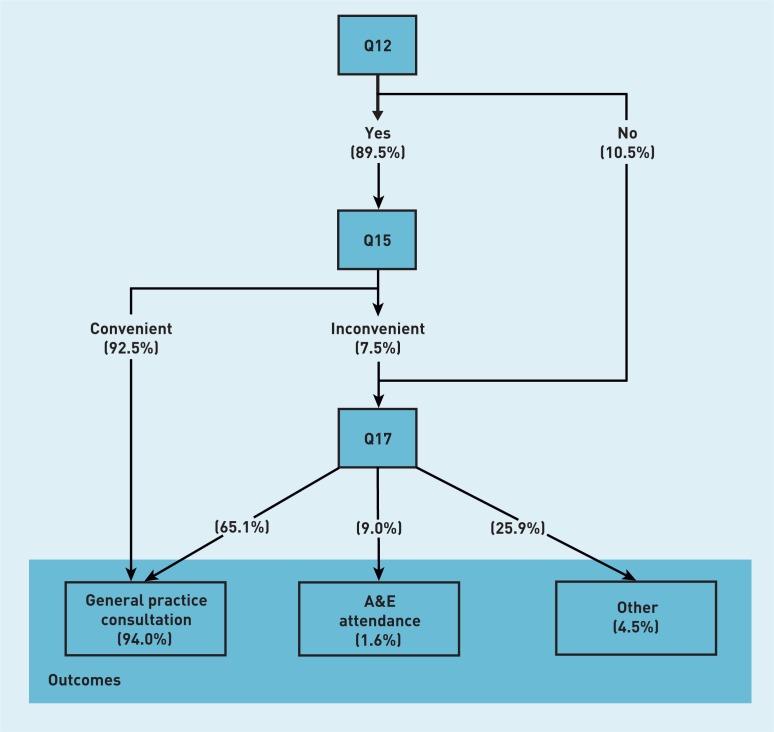
***Relative weighted frequencies of responses and branching structure of questions in the GP Patient Survey, 2012–2013.***

The analysis utilised the responses to three questions regarding a patient’s last attempt to see or speak to a GP or nurse from their general practice: ‘Were you able to get an appointment to see or speak to someone?’ (question 12); ‘How convenient was the appointment you were able to get?’ (question 15); and ‘What did you do on that occasion?’ (question 17). Whether responders were asked to complete questions 15 and 17 was dependent on their answers to questions 12 and 15 (Figure 1). Responses to question 12 were categorised as Yes (‘Yes’ or ‘Yes, but I had to call back closer to or on the day I wanted the appointment’) or No (‘No’); responses of ‘Can’t remember’ (3% of weighted total) were excluded from the calculation of the relative frequencies. Responses to question 15 were categorised as Convenient (‘Very convenient’ or ‘Fairly convenient’) or Inconvenient (‘Not very convenient’ or ‘Not at all convenient’). The response options for question 17 were grouped as: General practice consultation (‘Went to the appointment I was offered’, ‘Got an appointment for a different day’, or ‘Had a consultation over the phone’), A&E attendance (‘Went to A&E/a walk-in centre’), or Other (‘Saw a pharmacist’, ‘Decided to contact my surgery another time’, or ‘Didn’t see or speak to anyone’). Note that the use of the term ‘A&E department’ is inclusive of walk-in centres, in accordance with NHS England’s classification.^1^ The relative weighted frequencies of responses to each question were used to calculate the parameter estimate.

In Figure 1 all questions relate to the last time the responder wanted to see or speak to a GP or nurse from their general practice. There were 907 732 weighted responses to Q12. The relative weighted frequencies of answers to each question are given in parentheses, as percentages. Q13, Q14, and Q16 are omitted from Figure 1.

### Statistical methods

The final estimate was obtained by multiplying the two parameters defined above; its 99.9% confidence interval (CI) was calculated using the following formula (which incorporates the calculated variance for the product of two independent variables): X_1_*X_2_±3.29*√[(X_1_^2^+SE(X_1_)^2^)*(X_2_^2^+SE(X_2_)^2^)–(X_1_^2^*X_2_^2^)], where X_1_ and X_2_ are the two parameters described. The standard error and 99.9% CIs for the parameter relating to the number of general practice consultations were derived from the simple linear regression with financial year coded as a continuous variable; a quadratic term was not statistically significant when added to the regression equation to explore nonlinearity (*P* = 0.18). A bootstrap procedure with 500 000 repetitions was used to obtain the standard error and 99.9% CIs for the second parameter. The weighted GP Patient Survey results were first grouped into random subsamples of 100 responses. The relevant ratio was calculated for each subsample and then averaged, across subsamples, to obtain the ratio for each bootstrap sample; the CIs were derived from the observed distribution of these averaged ratios. A sensitivity analysis was conducted varying the number of consultations by ±12% (–12% is equal to the number of consultations previously estimated for 2008–2009) and the ratio parameter by ±25%. Data analysis was conducted using Stata MP (version 13.1).

## RESULTS

From 2000–2001 to 2008–2009, the estimated annual increase in the number of general practice consultations was 9.79 million (*P*<0.001; 99.9% CI = 7.04 to 12.5 million; *R^2^* = 0.98). The estimated number of general practice consultations in England in 2012–2013 was 345.6 million (99.9% CI = 331.5 to 359.7 million).

Ninety-four per cent of the weighted sample of survey responders indicated that a consultation was obtained on their last attempt; 88.0% of this group of responders had reported their appointment as convenient, while the remaining 12.0% had reported it as inconvenient or had initially indicated that they were unable to obtain an appointment. A small percentage of responders (1.6%) reported visiting an A&E department after being unable to obtain a convenient appointment. The bootstrapped mean ratio of relevant A&E attendances to general practice consultations was 0.0167 (99.9% CI = 0.0162 to 0.0172); for every 100 general practice consultations, 1.67 A&E attendances preceded by an unsuccessful appointment attempt are estimated to have occurred.

By multiplying the above values for each parameter (345.6 million consultations and 0.0167 relevant A&E attendances per general practice consultation), an estimated 5.77 million (99.9% CI = 5.49 to 6.05 million) A&E attendances were preceded by a patient being unable to obtain a convenient general practice appointment in England in 2012–2013. This equates to 26.5% of unplanned A&E attendances in this year (*n* = 21 738 637). The sensitivity analysis produced values between 3.81 million (17.5% of unplanned attendances) and 8.08 million (37.2%) A&E attendances (Table 1).

**Table 1:** Sensitivity analysis of estimated number of relevant A&E attendances in England in 2012–2013 (millions)

**Estimated number of general practice consultations in England, 2012–2013**	**Ratio of relevant A&E attendances to general practice consultations in GPPS sample, 2012–2013**				
	X_1_*0.75 0.01252	X_1_*0.9 0.01503	**X_1_ 0.01669**	X_1_*1.1 0.01836	X_1_*1.25 0.02087
**^a^303 900 000**	3.805	4.566	5.074	5.581	6.342
**X_2_345 600 000**	4.327	5.193	5.770^c^	6.347	7.212
**^b^387 300 000**	4.849	5.819	6.466	7.112	8.082

The unit of the estimates provided is millions of A&E attendances. X_1_: the bootstrapped mean ratio of relevant A&E attendances to general practice consultations in the GP Patient Survey (GPPS) sample (to 4 significant figures). X_2_: the estimated number of general practice consultations in England in 2012–2013, based on a simple linear regression of national data for 2000–2001 to 2008–2009^10^ (to 4 significant figures).

a The number of general practice consultations previously estimated for England in 2008–2009.^11^

b The number of general practice consultations estimated for 2012–2013 based on a doubling of the difference between 303.9 million and 345.6 million.

c 99.9% CI = 5.49 to 6.05 million.

## DISCUSSION

### Summary

The analysis suggests that a significant number of A&E attendances occurring in England each year are related to access to general practice. Although the percentage of attempts to obtain a general practice appointment that result in an A&E attendance appears to be small, the absolute effect is estimated to be large due to the considerable number of attempts to obtain appointments each year. Notably, the point estimate of 5.77 million A&E attendances corresponds to a substantially larger proportion of the unplanned A&E attendance workload than the total general practice workload (26.5% and 1.7% respectively in 2012–2013). Therefore, a change in utilisation patterns that may seem trivial in terms of demand for general practice services could have a considerable effect on A&E departments.

### Strengths and limitations

A national, validated patient survey was used to provide a novel estimate that is highly relevant to contemporary policy. However, the method does make several assumptions. First, it assumes that the linear trend in the annual number of general practice consultations observed between 2000–2001 and 2008–2009 continued through to 2012–2013. The method also assumes that a patient’s last attempt to obtain an appointment is representative of other attempts made in the year, and that patients’ propensities to subsequently visit an A&E department are independent of how often they attempt to obtain a general practice appointment. Furthermore, it is not possible to determine whether those patients who report subsequently visiting an A&E department had an objective clinical need to do so, or know the magnitude and direction of any possible bias in patients’ responses. This includes reporting bias, which could result from responders selecting answers they perceive to be of greater interest in the GP Patient Survey. Non-response bias could also be present if responding is influenced by factors unaccounted for by the weighting scheme used. Validation of the GPPS with data routinely recorded in general practices and A&E departments would be useful, to ascertain whether patients’ reported activity is consistent with administrative records. Moreover, since the GPPS only samples adults, the method assumes that the relative frequencies of outcomes of consultation attempts for children are equal to those for adults. The narrow 99.9% CIs demonstrate that any random error in the results obtained is small. While this analysis is exploratory, the results of the sensitivity analysis provide a range of values that do not alter the study conclusion: a large number of A&E attendances are likely to be preceded by unsuccessful attempts to obtain convenient general practice appointments in England each year.

### Comparison with existing literature

The estimates presented seem plausible when compared to other figures, including those obtained from studies of type 3 A&E departments (such as walk-in centres and urgent care centres^1^). For example, in a survey of 1886 patients attending 20 walk-in centres throughout England, 22% of responders registered to a general practice elsewhere had tried but were unable to obtain a convenient general practice appointment.^16^ At an urgent care centre co-located with an emergency department in London, England, 20% of surveyed patients attending with ‘minor illness’ had been unable to obtain a timely general practice appointment;^17^ 58% of patients had stated that one reason for their attendance was that it was ‘quicker than getting a GP appointment’,^17^ and the large majority of all attendees could have been managed by a GP or emergency nurse practitioner.^18^ A GP panel review of a random sample of 629 clinical case notes from patients attending an emergency department in Oxford, England, concluded that approximately 43% of patients could have been managed by a GP.^19^ Nationally, patients were recorded as receiving no treatment or advice only in 47% of A&E attendances and no investigation in 41% of attendances in 2012–2013.^2^

### Implications for research and policy

Future research could include a survey of a nationally representative sample of patients attending A&E departments of all types to provide a further estimate of the percentage that had tried but were unable to obtain a general practice appointment or a convenient appointment. Such research could also estimate the percentage of attendees that visited an A&E department without first contacting their general practice because they anticipated difficulties in obtaining a timely appointment, which it was not possible to do within this analysis.

Although a significant number of A&E attendances occurring in England each year are likely to be related to access to general practice, it does not necessarily follow that ensuring timely access will reduce the occurrence of A&E attendances. Additional demand for general practice appointments, which may or may not align with the unmet clinical needs of a population, could also be induced by such policy. These hypotheses require testing in further research employing high-quality designs. The existing literature on the effect of primary care interventions on A&E attendances is limited by low-quality study designs.^20^ However, the UK government’s pilot scheme of extended general practice opening hours provides an opportune natural experiment with which to examine this intervention’s effect on the occurrence of A&E attendances; it should be rigorously evaluated.
